# Surgical flow disturbances in dedicated minimally invasive surgery suites: an observational study to assess its supposed superiority over conventional suites

**DOI:** 10.1007/s00464-016-4971-1

**Published:** 2016-05-20

**Authors:** Mathijs D. Blikkendaal, Sara R. C. Driessen, Sharon P. Rodrigues, Johann P. T. Rhemrev, Maddy J. G. H. Smeets, Jenny Dankelman, John J. van den Dobbelsteen, Frank Willem Jansen

**Affiliations:** 1 Department of Gynecology, Leiden University Medical Center, PO Box 9600, 2300 RC Leiden, The Netherlands; 2 Department of BioMechanical Engineering, Technical University Delft, Mekelweg 2, 2628 CD Delft, The Netherlands; 3 Department of Gynecology, Bronovo Hospital, PO Box 96900, 2509 JH The Hague, The Netherlands

**Keywords:** Integrated operating room, Minimally invasive surgery, Technical equipment, Surgical flow disturbances, Video observation, Patient safety

## Abstract

**Background:**

Minimally invasive surgery (MIS) is frequently compromised by surgical flow disturbances due to technology- and equipment-related failures. Compared with MIS in a conventional cart-based OR, performing MIS in a dedicated integrated operating room (OR) is supposed to be beneficial to patient safety. The aim of this study was to compare a conventional OR with an integrated OR with regard to the incidence and effect of equipment-related surgical flow disturbances during an advanced laparoscopic gynecological procedure [laparoscopic hysterectomy (LH)].

**Methods:**

Using video recording, 40 LHs performed between November 2010 and April 2012 (20 in a conventional cart-based OR and 20 in an integrated OR) were analyzed by two different observers. Outcome measures were the number, duration and effect (on a seven-point ordinal scale) of the surgical flow disturbances (e.g., malfunctioning, intraoperative repositioning, setup device).

**Results:**

A total of 103 h and 45 min was observed. The interobserver agreement was high (kappa .85, *p* < .001). Procedure time was not significantly different (NS) [conventional OR vs. integrated OR, minutes ± standard deviation (SD), mean 161 ± 27 vs. 150 ± 34]. A total of 1651 surgical flow disturbances were observed (mean ± SD per procedure 40.8 ± 19.4 vs. 41.8 ± 15.9, NS). The mean number of surgical flow disturbances per procedure with regard to equipment was 6.3 ± 3.7 versus 8.5 ± 4.0, NS. No clinically relevant differences in the mean effect of these disturbances on the surgical flow between the two OR setups were observed.

**Conclusions:**

Performing LH in an integrated OR did not reduce the number of surgical flow disturbances nor the effect of these disturbances. Furthermore, in the integrated OR, repositioning of the monitors was a frequent and time-consuming source of disturbance. In order to maintain the high standard of surgical safety, the entire surgical team has to be aware that by performing surgery in an integrated OR different potential source for disruption arise.

In the era of rapidly evolving surgical techniques and technology, the patient, hospital, health insurance and government demand transparency in surgical outcomes and desire the highest degree of patient safety. Merely a decade ago, we started to accept the idea that surgical outcome is affected by more than the patient characteristics and skills of the surgeon alone [1]. In fact, the combination of patient risk factors, task complexity, individual surgical factors, and above all team functioning, operative events and operative environment are responsible for the outcome [1–3]. Especially in minimally invasive surgery (MIS), patient safety has to rely on a smooth course of the procedure and is depending on proper functioning of the equipment and the working environment [4]. Secondly, compared with open surgery, MIS is more prone to disruptions due to problems with the extensive amount of equipment it relies on (either presence, position or functioning) [4–8]. A systematic review revealed that on average per procedure three equipment-/technology-related errors occur. This resembles 23.5 % of the errors in the OR [9]. Additionally, they found that procedures that are more dependent on technology and/or equipment tended to show approximately three times higher equipment-related error rates [9]. Furthermore, during laparoscopic surgery, 47 % of the communication is equipment related, compared with 39 % during open surgery [10].

In order to guarantee an optimal working environment to perform MIS, the industry offers fully integrated surgical suites (e.g., ENDOALPHA by Olympus; iSuite by Stryker; OR1™ by Karl Storz). They state that—by their optimized design—these are the solution for efficient and safe surgical care by reducing operating room (OR) clutter and staff workload, increasing comfort and enhancing ergonomics and OR team performance [11–14]. Importantly, these statements are only describing potential benefits that are inherently biased by their manufactures and that are not based on objective research [12, 13, 15]. Regarding efficiency, only a couple of studies observed a small amount of time saving (i.e., ±4 min for setup and ±3 min for put away [13], ±6 min in ‘preanesthesia time’ [16] and ‘potentially’ ±6 min in overall OR time [11], respectively). Furthermore, a survey was performed under OR staff to investigate potential benefits of the integrated OR after 2 years of use. The results of the questionnaire showed a preference for the integrated OR; however, problems with staff education, integration and reliability were noted [17]. Another study explored the staff perceptions of the effects of an integrated OR on teamwork. The subjectively measured results of the nurses, consultants and trainees showed greater efficiency, better teamwork and reduced stress levels and therefore a strong preference for working in an integrated OR [18]. Although it is not clear whether an integrated OR is an useful, (cost-)effective and safe solution, globally many hospitals have invested or are investing in one or more integrated surgical suites [11, 17].

One could argue that an integrated OR facilitates such an improvement that patient safety is guaranteed and no extensive research is needed before applying this—expensive—technology [19]. However, it is well established that the failure of integrated devices also can lead to unforeseen problems, and from aviation technology, we know that even the smallest incidents can have catastrophic consequences [8, 20, 21]. One of the most striking examples is the crash of an airplane that, after a missed approach because of suspected gear nose malfunction, descended unnoticed because the entire flight crew became engrossed in the malfunction. Investigation revealed that only the nose landing gear position indicating system (i.e., the light bulb) was broken.

Therefore, quantitative research comparing equipment-related error rates in MIS performed in a conventional versus an integrated OR is desired. Studies describing surgical processes were generally based on live observation in the OR; video observation has only been used infrequently [6, 8, 22, 23]. Nevertheless, video registration is deemed superior since it is not limited by the capacity of an observer, cause-and-effect relationships are better analyzable, and the Hawthorne effect (i.e., the awareness of being observed alters the way a person behaves) is minimized [6, 7, 24, 25].

The aim of this prospective observational study was to compare a conventional OR with an integrated OR with regard to the incidence and effect of equipment-/instrument-related surgical flow disturbances during an advanced laparoscopic gynecological procedure (i.e., the laparoscopic hysterectomy (LH)).

## Materials and methods

In a university-affiliated teaching hospital (Bronovo Hospital, The Hague), a prospective registration study was set up to record and analyze surgical flow disturbances during the same procedure in two different OR settings. The LH was chosen as procedure under research, because it is an advanced laparoscopic procedure, performed by a dedicated operating team and requiring a wide array of endoscopic instruments and equipment. The study started in November 2010 and all consecutive LHs that were performed in the conventional (cart-based) OR were registered until the start of the construction of the new integrated OR (Karl Storz OR1™ integrated OR system, September 2011). After construction of the integrated OR (October 2011), the same amount of eligible procedures were registered in this setting. Based on a power calculation, we needed 16 procedures in each OR (average 8 ± 3 equipment-/instrument-related surgical flow disturbances per procedure and a reduction to 5 regarded to be achievable by the introduction of the integrated OR (power 80 %, type I error .05) [25]). The study design did not permit us to exactly determine the number of procedures beforehand, and furthermore, analysis of additional procedures would take an excessive amount of time. Therefore, it was strived for to acquire at least 15 and a maximum of 20 eligible procedures. All procedures were performed by one out of two gynecologists with more than 10 years of experience in advanced gynecologic laparoscopy and were assisted by a person who conducted a fellowship in MIS; a group of five alternated in the position of either circulating or scrub nurse. To become acquainted with the integrated OR setting, the entire operating team received multiple training sessions that were provided by the vendor.

In the conventional OR, all standard laparoscopic equipment (insufflator, light source and camera control unit, all manufactured by Karl Storz) was placed on a cart with one flat-screen high-definition monitor on top and one on a swivel arm. The electrosurgical equipment was placed on separate cart(s). In the integrated OR, the standard laparoscopic and electrosurgical equipment (manufacturers identical to conventional OR) was placed on a ceiling-mounted boom arm and three flat-screen high-definition monitors (of which one touch screen) were attached to separate ceiling-mounted boom arms.

**Fig. 1:**
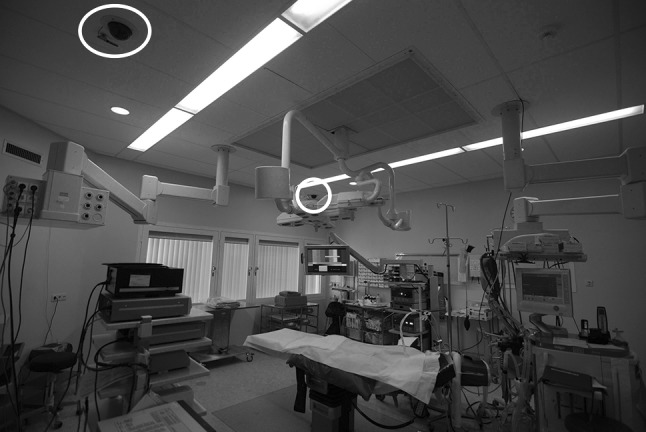
Conventional cart-based OR (dome cameras are *circled*)

To minimize the impact on the environment under study, the study was performed with video observation. The researcher (M.D.B.) was present in the OR at the start of each registration, but did not participate in the procedure. All procedures were recorded on a personal computer using a quad-audiovisual recording system that synchronously recorded the input from three video signals and four audio signals (MPEG Recorder 2.1, Noldus Information Technologies, Wageningen, The Netherlands). The video signals captured the endoscopic image and the image from two dome cameras that provided a room overview from different angles (one placed in a corner and one opposite in the middle of the long side of the operating room) (see Fig. 1). The audio signals were captured from two microphones placed on the ceiling next to the dome cameras and two wireless microphones placed on the surgical masks of the surgeon and scrub nurse, respectively. The recordings were started just before the time-out procedure and stopped after the skin of all port sites was sutured. In case technical problems related to the recording equipment were encountered, the procedure was excluded.

The study was approved by the Executive Board of the Bronovo Hospital. The recordings were only to be used for purpose of present study. Prior to the start of the study, all OR personnel was collectively informed about the study. They were told that the observations were performed to investigate the logistics of equipment and personnel during LH. From each patient, informed consent was obtained.

According to the methodology to analyze a peroperative surgical process described by Den Boer et al., all (potential) surgical steps that are commonly undertaken during LH were defined (Table 1) [26, 27]. The recordings were analyzed with The Observer^®^ XT 11.5 software (Noldus Information Technologies, Wageningen, The Netherlands). Two residents in Obstetrics and Gynecology (M.D.B. and S.R.C.D.) observed the recordings. A random sample of six recordings was scored by both observers. The findings of the two observers for these six procedures were compared, and the interobserver agreement was calculated (function incorporated in The Observer^®^ XT 11.5 software). If satisfactory interobserver agreement would be achieved, the remaining procedures could be annotated by the two observers separately (randomly allocated and analyzed in a non-chronological random order) [5, 23].

**Table 1:** Surgical phases and (potential) surgical steps commonly undertaken during laparoscopic hysterectomy (adjusted from Den Boer et al. [26])

Surgical phases	Surgical steps
1. Preoperative	
	1.1. OR ready (clean, air quality, pressure)
	1.2. Instruments and devices present and functioning
	1.3. Patient to OR
	1.4. Patient on OR table
	1.5. Time-out procedure
	1.6. Position patient on OR table
	1.7. Team scrubs in washing room
	1.8. Sterile preparation of instruments
2. Anesthesia and surgical preparation	
	2.1. Anesthesia and intubation
	2.2. Sterilization operating area
	2.3. Draping the patient
	2.4. Insert urine catheter
	2.5. Insert mobilizer in uterus
	2.6. Install instruments
3. Procedure	
3.1. Create CO2 pneumoperitoneum	3.1.1. First incision and insert Veress or Hasson
	3.1.2. Insufflate the abdomen
3.2. Insert access ports	3.2.1. Insert first (optical) port
	3.2.2. Insert laparoscope
	3.2.3. Inspect abdomen (active bleeding, 360 look, operatability)
	3.2.4. Insert second port under direct sight
	3.2.5. Inspect and judge operatability/unexpected pathology)
	3.2.6. Insert third port under direct sight
	3.2.7. Insert fourth port under direct sight
3.3. Preparation operative area	3.3.1. Dissect adhesions to uterus/ovaria/intestine in pelvis
	3.3.2. Mobilize intestine out of pelvis
3.4. Expose uterine arteries	3.4.1. Dissect ligaments and mobilize uterus
	3.4.2. Skeletonized uterine arteries
	3.4.3. Push off bladder
	3.4.4. Identify location of ureters
3.5. Transect uterine arteries	3.5.1. Transect left uterine artery
	3.5.2. Transect right uterine artery
	3.5.3. Check color of uterus
	3.5.4. Check if bladder and arteries are skeletonized enough
3.6. Separate uterus from vagina	3.6.1. Colpotomy
	3.6.2. Pneumoperitoneum is lost
3.7. Specimen retrieval	3.7.1. Morcellated uterus
	3.7.2. Extract uterus through vagina
3.8. Closure of the vaginal cuff	3.8.1. Insert needle
	3.8.2. Suture vaginal cuff
	3.8.3. Extract needle
3.9. Final check and irrigation	3.9.1. Check hemostasis
	3.9.2. Check vaginal cuff stump
3.10. Close-up patient	3.10.1. Remove instruments
	3.10.2. Remove accessory operating ports (under direct sight)
	3.10.3. Check access wounds/bleeding
	3.10.4. Release CO2 from abdomen
	3.10.5. Remove laparoscope and first trocar port
	3.10.6. Suture port wounds
	3.10.7. Remove draping
4. Extubation	
	4.1. Patient awake
	4.2. Extubation
5. Postoperative	
	5.1. Patient from OR table to ward bed
	5.2. Sign-out procedure
	5.3. Bring patient to recovery
6. Interoperative	
	6.1. Cleaning of the OR

### Annotation and statistics

From each procedure, the predefined surgical steps and the presence and effect of predefined surgical flow disturbances were annotated (Table 1). Surgical flow disturbances were defined as stimuli (potentially) distracting one or more members of the sterile team (Table 2). To assess the (potential) severity, the effect on the sterile team members caused by each observed surgical flow disturbance was graded according to a seven-point ordinal scale modified by Persoon et al. (originally described by Healey et al.) (Table 3) [25, 28]. This scale ranges from ‘1’ as a potentially distracting stimulus to ‘7’ when the sterile team’s work is completely interrupted. Primary outcome measures were the number of surgical flow disturbances per procedure. Secondary, a qualitative assessment was made comparing the types, effect and duration of these surgical flow disturbances for the two different OR settings.

**Table 2:** Observed types of surgical flow disturbances

Equipment-/instrument-related	Setup device/connection
	Intraoperative repositioning
	Malfunctioning
	Not present
	Sterility
	Other/unclear
Environmental	Pager/telephone
	Door washing room
	Radio use
Personnel-related	Communication failure
	Irrelevant conversation
Procedure-related	Extra coagulation bleeding-site
	Unexpected adhesions
	Limited vision (condensation/smoke)
	Adverse event
	Conversion to laparotomy

**Table 3:** Effect of observed surgical flow disturbances (according to Persoon et al. [25])

1. Events with the potential to distract the sterile team
2. Sterile team member momentarily distracted: possible involvement of a single sterile member in an event not related to the primary task, e.g., a short head turn in response to a visual or auditory stimulus
3. Sterile team member engages in distraction: similar distraction in 2, but the sterile member engages with the source of distraction by verbally responding while maintaining primary task activity (multitasking)
4. Sterile team member’s primary task interrupted: a single team member ceases his/her current tasks to engage entirely in the distracting stimulus
5. Sterile team momentarily distracted: two or more sterile team members respond to a stimulus with a short head turn, no verbal response
6. Sterile team engage in secondary tasks: two or more team members engage with the source of distraction by verbally responding while maintaining primary task activity
7. Sterile team’s work interrupted—operation flow disrupted: interruption of the current primary task of the sterile team, the operation flow is disrupted

Patient and procedure characteristics were derived by chart review. For statistical analysis, The Observer^®^ XT 11.5 software and SPSS 20.0 statistical software (Chicago, IL, USA) were used. A Pearson Chi-square test was used to compare proportions, and a Student’s *t* test was used for continuous variables. To describe non-normally distributed data (kurtosis between −1 and +2) or in case Levene’s test showed no homogeneity of variance, the median and interquartile range (IQR, 25th and 75th percentiles) were used and a Mann–Whitney test was performed. A *p* < .05 was considered statistically significant.

## Results

**Fig. 2:**
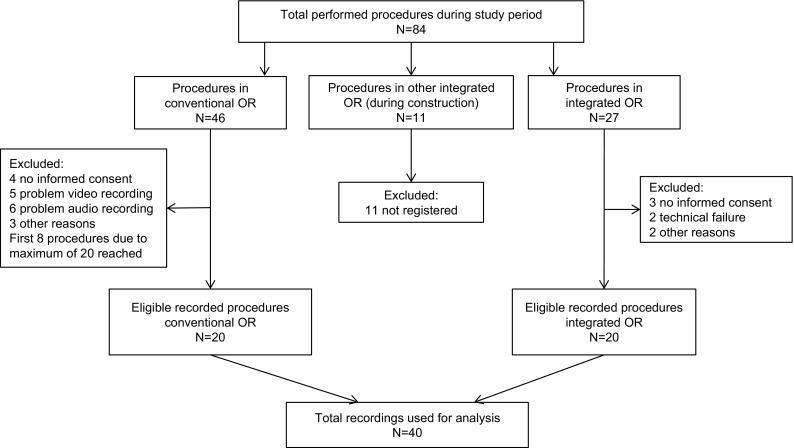
Inclusion of eligible procedures

During the study period, 46 LHs were performed in the conventional OR. Of those, 18 were not eligible (4 were not recorded because of no consent, 5 were excluded because of problems with the video recording, 6 due to audio problems and 3 for other reasons). In order to obtain the predefined 20 most recent procedures, first 8 procedures that were recorded were not observed. During construction of the operating room that was equipped with the observation system, 11 LHs were performed in another integrated OR. Subsequently, in the observational integrated OR 27 LHs were performed until 20 LHs that were registered were eligible (3 were not recorded because of no consent, 2 were excluded because of technical problems and 2 for other reasons) (Fig. 2).

The overall observation duration of these 40 procedures was 103 h and 45 min. Patient and procedure characteristics were similar between the two OR settings (Table 4). Only 3 minor complications were noted, all postoperatively (Table 5). Procedure time (conventional OR vs. integrated OR, minutes ± standard deviation, mean 161 ± 28 vs. 150 ± 34) and operating time (skin to skin, mean 126 ± 27 vs. 116 ± 31) were not significantly different (NS) (Table 6).

**Table 4:** Patient and procedure characteristics of analyzed LHs performed in the Bronovo Hospital, The Hague, between January 2011 and April 2012

	Conventional OR (*N* = 20)		Integrated OR (*N* = 20)		*p* value
	Median	IQR	Median	IQR	
Age (years)	48.2	43.9–55.2	47.1	43.5–56.0	.850^a^
BMI (kg/m^2^)	24.9	22.7–27.3	25.3	22.5–28.9	.871^a^
Uterine weight (gram)	165	97–256	149	107–208	.643^a^
Operating time (minutes)^b^	122	±31	124	±36	.816^c^
Estimated Blood loss (mL)	100	50–175	75	50–150	.702^a^
Hospital stay (days)	2.0	1.1–2.1	1.9	1.3–2.0	.795^a^
Benign indication (%)	70.0 %		55.0 %		.514^d^

*IQR* Inter quartile range (25th and 75th percentile), *BMI* body mass index

^a^Mann–Whitney test

^b^Time according to medical file

^c^Mean ± standard deviation and Student’s *t* test because of normal distribution

^d^Pearson’s Chi-square

**Table 5:** Adverse events all analyzed LHs. All adverse events did not require re-operation and occurred postoperatively

	Conventional OR (*N* = 20)	Integrated OR (*N* = 20)	Overall (*N* = 40)
Infection	1^a^ (5.0 %)	0	1 (2.5 %)
Blood loss > 1L	0 (0 %)	1^b^ (5.0 %)	1 (2.5 %)
Others	1^c^ (5.0 %)	0	1 (2.5 %)
Total	2 (10.0 %)	1 (5.0 %)	3 (7.5 %)

^a^Urinary tract infection

^b^Postoperative drop in hemoglobin. CT scan showed approximately 1500 cc free fluid intraabdominally. Vital signs were stable, and after a blood transfusion with 2 packed cells, hemoglobin remained stable

^c^Patient suffered from sensibility loss in her right hand. The neurologist diagnosed a neuropraxia of the median nerve. Conservative management resulted in almost complete recovery

**Table 6:** Durations of all analyzed LHs (in minutes:seconds)

Observation duration	Conventional OR (*N* = 20)			Integrated OR (*N* = 20)			Total (*N* = 40)
	53 h:42:55			50 h:02:38			103 h:45:33
	Mean	±SD	Min–Max	Mean	±SD	Min–Max	*p* value^c^
Procedure time^a^	161:09	±27:38	107:37–210:24	150:08	±34:09	98:24–214:52	.269
Operating time^b^	126:17	±26:35	66:20–175:44	115:42	±30:38	71:48–174:58	.251

^a^Time between patient entering OR and leaving OR

^b^Time between first incision and last suture (skin to skin)

^c^Unpaired *t* test calculated using http://www.graphpad.com/quickcalcs/ttest1/?Format=SD

In all six observations, both observers showed excellent agreement in their annotations (Cohen’s kappa of .79–.98, all observations combined .85, *p* < .001). Therefore, the remaining procedures were annotated by the two observers separately (in total 36 observations by M.D.B. and 10 by S.R.C.D., respectively).

In total, during all 40 procedures, the researcher was present in the OR for 115 min (82 min in the conventional OR and 32 min in the integrated OR) [1.9 % of total observation time, mean 4 min per procedure, 0–12 (min–max)]. The mean effect on the sterile team members of this presence was 1.7 (see Table 3). The mean effect of noticed study awareness was 3.6 (*N* = 52 in 40 procedures).

### Incidence and effect of surgical flow disturbances

A total of 1651 surgical flow disturbances were scored (mean ± SD per procedure 40.8 ± 19.4 vs. 41.8 ± 15.9, NS) (unless otherwise specified, all comparisons are conventional vs. integrated OR). With regard to equipment, the mean number of surgical flow disturbances per procedure (setup of device, disturbance or problem regarding equipment, and intraoperative repositioning) was 6.3 ± 3.7 versus 8.5 ± 4.0, NS. More specifically, the mean duration of surgical flow disturbances regarding the setup of devices [*N* = 16 (total number of disturbances in 20 procedures), 1:16 ± 2:05 (mean ± SD in minutes:seconds) vs. *N* = 27, 1:57 ± 4:32, NS], disturbances or problems regarding equipment in general (*N* = 93, 2:19 ± 3:50 vs. *N* = 110, 1:54 ± 2:19, NS) and intraoperative repositioning (*N* = 16, 0:45 ± 0:37 vs. *N* = 33, 0:39 ± 0:32, NS) did not significantly differ either. Similarly, the mean effect of these disturbances did not show a clinically relevant difference (setup: 5.3 ± 1.6 vs. 4.2 ± 2.0, NS; disturbances regarding equipment in general: 5.8 ± 1.7 vs. 5.3 ± 1.8, *p* = .04; intraoperative repositioning: 4.6 ± 1.9 vs. 4.1 ± 1.7, NS).

**Fig. 3:**
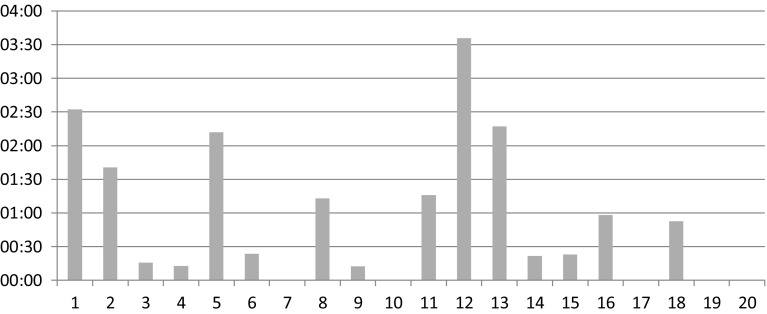
Duration (minutes:seconds) of intraoperative repositioning of a monitor in the integrated OR (per procedure, chronological order)

The number and total duration of the different devices and instruments accountable for these disturbances are shown in Table 7. Particularly, the difference between the conventional OR and the integrated OR with respect to disturbances caused by ‘monitor’ is notable (*N* = 10, total duration 18 min vs. *N* = 46, 87 min; mean effect 4.7 ± 2.2 vs. 4.1 ± 1.7, NS). In the conventional OR one disturbance was caused by a failing connection of the second monitor (lasting 11 min). In the integrated OR during four procedures there were problems with activating the third monitor (which was eventually found out to be caused by a hardware problem) (total duration 64 min). The majority of the remaining duration of the surgical flow disturbances regarding the monitor in the integrated OR were caused by intraoperative repositioning (*N* = 28, 18 min, mean effect 4.1). A chronological representation per procedure is shown in Fig. 3.

**Table 7:** Devices and instruments accountable for surgical flow disturbances with respect to setup of device, disturbance or problem in general, and intraoperative repositioning

	Conventional OR (*N* = 20)		Integrated OR (*N* = 20)	
Surgical flow disturbance regarding	*N*	Total duration (h:min:sec)	*N*	Total duration (h:min:sec)
Devices				
Diathermy	27	00:46:36	30	00:59:00
Endoscope	2	00:01:00	3	00:17:11^a^
Insufflator	19	00:21:07	21	00:17:34
Irrigation suction	7	00:09:15	9	00:05:44
Light source	3	00:00:50	4	00:02:24
Morcellation device	1	00:03:55	4	00:04:54
Pedals	–		6	00:04:33
Instruments				
Instruments—dismountable	25	01:52:38	20	00:45:06^b^
Instruments—non-dismountable	11	00:19:04	13	00:25:34
Trocar	3	00:01:39	1	00:00:53
Devices—OR-related				
Monitor	10	00:17:52	46	01:26:35^c^
Overhead light	3	00:00:52	2	00:00:49
Table	6	00:05:05	7	00:11:18
Tower	11	00:09:16	6	00:05:14

^a^Difference in total duration caused by one event lasting 16 min

^b^Difference in total duration caused by a variety of non-OR-related problems

^c^Mean degree of influence 4.7 ± 2.2 versus 4.1 ± 1.7, *p* = .37

The difference in total duration for surgical flow disturbances regarding ‘instruments—dismountable’ is caused by a variety of non-OR-related problems. No difference was found with regard to the number of surgical flow disturbances caused by devices that were not present in the OR [*N* = 12, 2:27 ± 2:00 (mean ± SD in minutes:seconds) vs. *N* = 16, 3:31 ± 2:37, NS].

## Discussion

The number of equipment-related surgical flow disturbances is not reduced by performing laparoscopic hysterectomy in an integrated OR instead of a conventional cart-based OR. Similarly, regarding the effect of these disturbances on the sterile team members, no clinically relevant difference between the two types of OR was found. Moreover, in the integrated OR, intraoperative repositioning of the monitors is a frequent and time-consuming source of disturbance.

It has been stated that optimizing the operating environment potentially may have a more significant impact on overall surgical outcome than improving individual surgical skill [29]. Although our study was not designed to detect differences in surgical outcome, we found that an integrated OR, as one of the most promising solutions to improve the operating environment, did not result in a reduction in equipment-related surgical flow disturbances. As a matter of fact, we even identified some potential hazards with the introduction of an integrated OR. The increased occupation that we observed with the repositioning of the monitors is important and has also been recognized by others [8]. Due to limitations in the degrees of freedom of the monitor and the ceiling-mounted boom arm, these disturbances were relatively time-consuming. Obviously, precise placement of the monitors can optimize the posture and improve ergonomics of all members of the surgical team [30]. However, apparently, the surgical team does not seem to be fully aware of the potential negative effect on the procedure during the repositioning. Having said this, the repositioning of the monitors fortunately did not have a direct effect on patient safety. However, what it does imply is that all implementations of either new technology, devices or instruments could potentially be hazardous in the chain of patient safety, because, especially during implementation of a new tool, one has to be aware that these are not always intuitive or straightforward in use [5]. Furthermore, the complete integration of the devices prevents easy (intraoperative) replacement in case of a dysfunctional device. Therefore, in an integrated OR, monitor positioning should be carefully planned and prepared preoperatively. This could be realized by the incorporating this as a mandatory item in a preoperative checklist [5, 31].

Previous research has demonstrated that surgical flow disturbances are directly related to surgical performance [25, 32, 33]. The number of surgical flow disturbances per procedure that we objectified was in line with similar studies. Persoon et al. [25] described surgical flow disturbances during endourological procedures (median operating time 35 min) and found a median of 20 disturbances per procedure of which 1.7 were equipment related. Moreover, also the effect of these disturbances on the sterile team was similar to our results (4.97 vs. 4.1–5.8). Furthermore, Verdaasdonk et al. [8] observed problems with equipment during laparoscopic cholecystectomy. In 30 procedures, they identified 58 disturbances. Since laparoscopic cholecystectomy is usually performed in approximately an hour and in general is being considered as one of the lesser advanced procedures in surgery, this rate seems also comparable to the 6.3–8.5 equipment-related disturbances we found. Nevertheless, although it is known that laparoscopic surgery is prone to instrument-related disturbances [9], this number leaves substantial room for improvement, and apparently this needs to be realized by other solutions than performing minimal invasive surgery in an integrated OR instead of a conventional OR.

As recommended by others, taking care of a structured implementation process is a key factor for an innovation to become a success [5, 34, 35]. During the construction period, the complete OR team received multiple training sessions by the vendor to become familiar with the new OR setting. Despite this, and beside the repositioning-related disturbances caused by the monitor, we incidentally observed some struggling with the new equipment. This finding could be attributable to the learning curve. Regardless of training, in daily practice every new technique and technology comes along with a period a time during which one has to become completely familiar with the new environment. However, in our opinion, if the integrated OR really could reduce the number of surgical flow disturbances, that should—at least partially—be measurable from the first procedure performed in this OR, from both a patient safety and an ethical perspective. Moreover, observing 20 procedures in both types of OR should be sufficient to detect a clinically relevant difference, and graphical representations of our results did not show a learning curve (e.g., Fig. 3).

One of the strengths of our study was the use of video observation making rewinding and playing again possible, in order to make sure all disturbances and their consequences are accurately interpreted. As a consequence, also the presence and influence of the researcher during the procedure and the awareness of the OR team on the study was reduced to a minimum, thereby making the interference of the study with its own results (the Hawthorne effect) negligible.

Despite this strength for research purposes, video observation is also limited by both the very time-consuming analysis and legal aspects. These downsides still have to be overcome, before it can become common practice for research as well as training and legal purposes [8, 24, 36]. In our opinion, a more widespread adoption of video recording has an enormous potential to improve quality and safety of surgery. It could be used for general reviewing of the procedural steps, but mainly for the analysis of (near) failures and (team) training purposes, thereby taking quality improvement to the next level [37]. Finally, also patients were positive about the idea of having their procedures recorded [38].

The presence of equipment-related surgical flow disturbances remains multifactorial. The proclaimed reduction in these disturbances during MIS in an integrated OR could not be shown. Especially with respect to MIS, a dedicated training has been proven to result in increased safety, shorted operating time and less conversions [39]. Also a dedicated (nurse) team is beneficial to patient safety [40]. Furthermore, of all types of disturbances, equipment problems have among the highest influence on the surgical flow and procedures during which disruptions occur take longer. Therefore, it may be assumed that a well-trained and dedicated surgical team will be more beneficial to patient safety than changing the OR setting, i.e., performing MIS in an integrated OR instead of a conventional cart-based OR [4, 41, 42].

Nevertheless, the integrated OR does have already proven advantages that we did not take into account in our study. Most importantly, for all team members the ergonomics are more favorable, thereby reducing physical complaints and eventually dropout [30]. Furthermore, also time saving in the preoperative setup has been observed [11, 13, 16]. Therefore, performing MIS in an integrated OR could be regarded an ergonomically responsible innovation for those who are frequently performing advanced MIS.

In conclusion, compared to a conventional OR, performing MIS in an integrated OR does not seem to increase patient safety either by a reduction in the number of surgical flow disturbances or by a reduction in the effect of these disturbances on the members of the sterile team. In order to maintain the high level of surgical safety that has been established by laparoscopic surgery, the entire surgical team has to be fully aware that by performing surgery in an integrated OR different potential source for disruption arise.
